# Structural Alteration of Gut Microbiota during the Amelioration of Human Type 2 Diabetes with Hyperlipidemia by Metformin and a Traditional Chinese Herbal Formula: a Multicenter, Randomized, Open Label Clinical Trial

**DOI:** 10.1128/mBio.02392-17

**Published:** 2018-05-22

**Authors:** Xiaolin Tong, Jia Xu, Fengmei Lian, Xiaotong Yu, Yufeng Zhao, Lipeng Xu, Menghui Zhang, Xiyan Zhao, Jian Shen, Shengping Wu, Xiaoyan Pang, Jiaxing Tian, Chenhong Zhang, Qiang Zhou, Linhua Wang, Bing Pang, Feng Chen, Zhiping Peng, Jing Wang, Zhong Zhen, Chao Fang, Min Li, Limei Chen, Liping Zhao

**Affiliations:** a Guang’anmen Hospital, China Academy of Chinese Medical Sciences, Beijing, People’s Republic of China; b State Key Laboratory of Microbial Metabolism, School of Life Sciences and Biotechnology, Shanghai Jiao Tong University, Shanghai, People’s Republic of China; c Graduate School, Beijing University of Chinese Medicine, Beijing, People’s Republic of China; d Shanghai Centre for Systems Biomedicine, Shanghai Jiao Tong University, Shanghai, People’s Republic of China; uBiome; New York University

**Keywords:** clinical trial, gut microbiota, hyperlipidemia, metformin, traditional Chinese medicine, type 2 diabetes

## Abstract

Accumulating evidence implicates gut microbiota as promising targets for the treatment of type 2 diabetes mellitus (T2DM). With a randomized clinical trial, we tested the hypothesis that alteration of gut microbiota may be involved in the alleviation of T2DM with hyperlipidemia by metformin and a specifically designed herbal formula (AMC). Four hundred fifty patients with T2DM and hyperlipidemia were randomly assigned to either the metformin- or AMC-treated group. After 12 weeks of treatment, 100 patients were randomly selected from each group and assessed for clinical improvement. The effects of the two drugs on the intestinal microbiota were evaluated by analyzing the V3 and V4 regions of the 16S rRNA gene by Illumina sequencing and multivariate statistical methods. Both metformin and AMC significantly alleviated hyperglycemia and hyperlipidemia and shifted gut microbiota structure in diabetic patients. They significantly increased a coabundant group represented by *Blautia* spp., which significantly correlated with the improvements in glucose and lipid homeostasis. However, AMC showed better efficacies in improving homeostasis model assessment of insulin resistance (HOMA-IR) and plasma triglyceride and also exerted a larger effect on gut microbiota. Furthermore, only AMC increased the coabundant group represented by *Faecalibacterium* spp., which was previously reported to be associated with the alleviation of T2DM in a randomized clinical trial. Metformin and the Chinese herbal formula may ameliorate type 2 diabetes with hyperlipidemia via enriching beneficial bacteria, such as *Blautia* and *Faecalibacterium* spp.

## IMPORTANCE

Metabolic diseases such as T2DM and obesity have become a worldwide public health threat. Accumulating evidence indicates that gut microbiota can causatively arouse metabolic diseases, and thus the gut microbiota serves as a promising target for disease control. In this study, we evaluated the role of gut microbiota during improvements in hyperglycemia and hyperlipidemia by two drugs: metformin and a specifically designed Chinese herbal formula (AMC) for diabetic patients with hyperlipidemia. Both drugs significantly ameliorated blood glucose and lipid levels and shifted the gut microbiota. *Blautia* spp. were identified as being associated with improvements in glucose and lipid homeostasis for both drugs. AMC exerted larger effects on the gut microbiota together with better efficacies in improving HOMA-IR and plasma triglyceride levels, which were associated with the enrichment of *Faecalibacterium* spp. In brief, these data suggest that gut microbiota might be involved in the alleviation of diabetes with hyperlipidemia by metformin and the AMC herbal formula.

## INTRODUCTION

Type 2 diabetes mellitus (T2DM), a global epidemic disease with a rapid growth in rates of incidence and prevalence, affects people from all income levels worldwide (1). It is a metabolic disease characterized by insulin resistance and β-cell dysfunction (2) associated with inflammation caused by overnutrition and other environmental factors (3–5). The intestinal microbiota has been proposed to play a vital role in the pathophysiology of obesity, T2DM, and other metabolic diseases (6–8). Transplantation of the gut microbiota from the obese human gut can transfer the obese phenotypes to germfree mice, indicating the causative role of gut microbiota in the development of obesity and metabolic diseases (7). Moreover, an opportunistic pathogen, Enterobacter cloacae B29, isolated from a diabetic obese human’s gut, can induce obesity and insulin resistance in germfree mice (9). These findings disclosed that gut microbiota can causatively arouse insulin resistance, obesity, and T2DM. Furthermore, a more recent study showed that dietary modulation of gut microbiota contributes to alleviation of both genetic and simple obesity in children (10). Therefore, targeting the gut microbiota with prebiotics, probiotics, diets, and drugs would be a novel therapeutic approach to ameliorate diabetes, hyperlipidemia, and other metabolic diseases.

Metformin is one of the most widely used oral antihyperglycemic drugs recommended as the first-line remedy for the treatment of diabetes. It also shows potential applications in the treatment or prevention of hyperlipidemia (11, 12) and cardiovascular diseases (13). Numerous studies showed that metformin might function by inhibition of hepatic gluconeogenesis via activation of the AMP-activated protein kinase (AMPK) signaling pathway (14, 15). However, in contrast to oral dosing, intravenously administered metformin does not improve glucose metabolism (16), indicating that gut microbiota might be the target. A more recent study demonstrated that metformin enriched the *Akkermansia* sp. population in high-fat-diet (HFD)-induced hyperglycemic and hyperlipidemic mice. Moreover, an inoculum of *Akkermansia* administered to diabetic mice exhibited a similar glucose-lowering efficacy to metformin, suggesting that alteration of gut microbiota is a new mechanism of metformin’s therapeutic effect in treatment of diabetes (17). However, our study showed that metformin significantly increased short-chain fatty acid (SCFA)-producing bacteria such as *Blautia* spp. and decreased *Akkermansia* spp., together with the amelioration of obesity in HFD-induced rats (18). As for in humans, when using metagenomic data from a multicountry T2DM metagenomic data set, univariate tests showed a significant increase in *Escherichia* spp. and a reduced abundance of *Intestinibacter* spp. in T2DM metformin-treated samples (19). However, this was the analysis of a collected microbial data set rather than a metformin-mediated gut microbiota shift analysis in a randomized clinical trial. Therefore, investigations with randomized clinical trials are needed to better understand metformin’s effect on gut bacteria in humans.

Traditional Chinese medicine (TCM) is a form of polypharmacy that has been developed and advocated for the treatment of many diseases for over 2,500 years in China. Our previous study suggested that gut microbiota might be involved in the effect of a standardized traditional herbal formula, GQD, which indicated that TCM can also modulate gut microbiota structure as a potential mechanism for improvement of diseases (20). An herbal formula consisting of eight herbs (AMC) was specifically developed for type 2 diabetic patients with hyperlipidemia. The herbs used in this formula, such as *Rhizoma Anemarrhenae* (21), Momordica charantia (22, 23), Coptis chinensis (24), Aloe vera (25, 26), and red yeast rice (27, 28), have profound effects in ameliorating blood glucose and lipid levels in various clinical trials and animal experiments. In addition, this herbal formula has been used in clinical application for more than 10 years, which displayed satisfying efficacy. AMC contains the herb Coptis chinensis, whose major component, berberine, has been clinically demonstrated to be able to alleviate diabetes with hyperlipidemia, possibly via increasing SCFA-producing bacteria as shown in rats with HFD-induced obesity (29). We hypothesized that gut microbiota might also be involved in the efficacy of AMC.

To test the hypothesis that alteration of gut microbiota may be involved in the alleviation of T2DM with hyperlipidemia by metformin and AMC, 450 patients with T2DM and hyperlipidemia were randomly assigned to ether a metformin- or AMC-treated group. After 12 weeks of treatment, 100 patients were randomly selected from each group and assessed for clinical improvements. The effects of the two drugs on the intestinal microbiota were evaluated by analyzing the V3 and V4 regions of the 16S rRNA gene by Illumina sequencing and multivariate statistical analysis.

## RESULTS

### Alleviation of hyperglycemia and hyperlipidemia by metformin and AMC.

**FIG 1:**
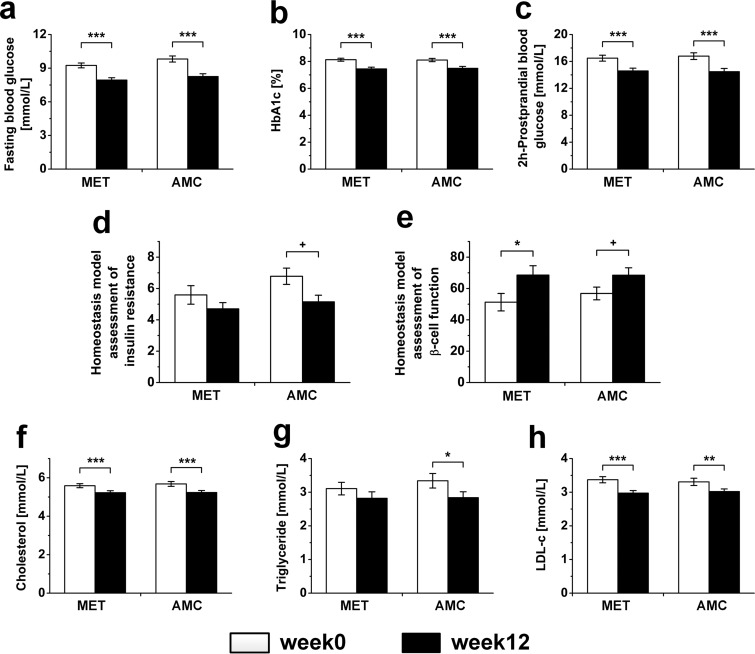
Both metformin and AMC herbal formula significantly improved glucose and lipid homeostasis of T2DM patients with hyperlipidemia. (a) Fasting blood glucose. (b) HbA1c. (c) Two-hour postprandial blood glucose. (d) Homeostasis model assessment of insulin resistance (HOMA-IR). (e) Homeostasis model assessment of β-cell function (HOMA-β). (f) Cholesterol. (g) Triglyceride. (h) LDL-c. Data are presented as means ± standard errors of the means (SEM). MET, metformin; AMC, AMC herbal formula. ***, *P* < 0.0001, **, *P* < 0.001, *, *P* < 0.01, and +, *P* < 0.05, by two-tailed paired *t* test.

We analyzed the clinical data from 200 participants as shown in Table S2 in the supplemental material. The baseline variables showed no significant differences between the two groups. After 12 weeks of treatment, both metformin and AMC significantly improved fasting blood glucose (FBG), glycosylated hemoglobin (HbA1c), 2-h postprandial blood glucose (PBG), and homeostatic model assessment of β-cell function (HOMA-β). However, only AMC significantly improved homeostatic model assessment of insulin resistance (HOMA-IR), although no significant difference was obtained between AMC and metformin by analysis of covariance (ANCOVA). There were no significant differences between the two treatment groups in the alleviation of diabetes, except that insulin resistance was only significantly improved by AMC (Fig. 1a to e; Table S2). Besides, the AMC herbal formula significantly reduced total plasma cholesterol, triglyceride, and low-density lipoprotein cholesterol (LDL-c) levels, body weight, body mass index (BMI), and waist and hip circumferences of diabetic patients with hyperlipidemia (Fig. 1f to h; see Fig. S2a to d and Table S2 in the supplemental material). These parameters were also significantly ameliorated by metformin, except for triglyceride levels. However, metformin significantly increased diastolic blood pressure unexpectedly, which was significantly higher than that by AMC according to ANCOVA (Fig. S2e and f).

10.1128/mBio.02392-17.2 FIG S1  The AMC herbal formula. (a) Photos of the boil-free granules of 8 herbs in the AMC herbal formula. (b) HPLC fingerprinting of AMC herbal formula. (c) Eight main chemicals in AMC. Download FIG S1, TIF file, 1.5 MB. Copyright © 2018 Tong et al. 2018 Tong et al. This content is distributed under the terms of the Creative Commons Attribution 4.0 International license.

10.1128/mBio.02392-17.3 FIG S2  The effect of metformin and AMC herbal formula on body weight and blood pressure of T2D patients with dyslipidemia. MET, metformin; AMC, AMC herbal formula. ***, *P* < 0.0001, and *, *P* < 0.05, using the paired *t* test; ^+^, *P* < 0.05 using ANCOVA. Data are presented as means ± SEM. Download FIG S2, TIF file, 0.4 MB. Copyright © 2018 Tong et al. 2018 Tong et al. This content is distributed under the terms of the Creative Commons Attribution 4.0 International license.

10.1128/mBio.02392-17.7 TABLE S1  Composition of the AMC herbal formula. Download TABLE S1, DOCX file, 0.1 MB. Copyright © 2018 Tong et al. 2018 Tong et al. This content is distributed under the terms of the Creative Commons Attribution 4.0 International license.

10.1128/mBio.02392-17.8 TABLE S2  Demographic, baseline, and study endpoint characteristics of clinical parameters. Download TABLE S2, DOCX file, 0.1 MB. Copyright © 2018 Tong et al. 2018 Tong et al. This content is distributed under the terms of the Creative Commons Attribution 4.0 International license.

### Alteration of gut microbiota structure by metformin and AMC.

**FIG 2:**
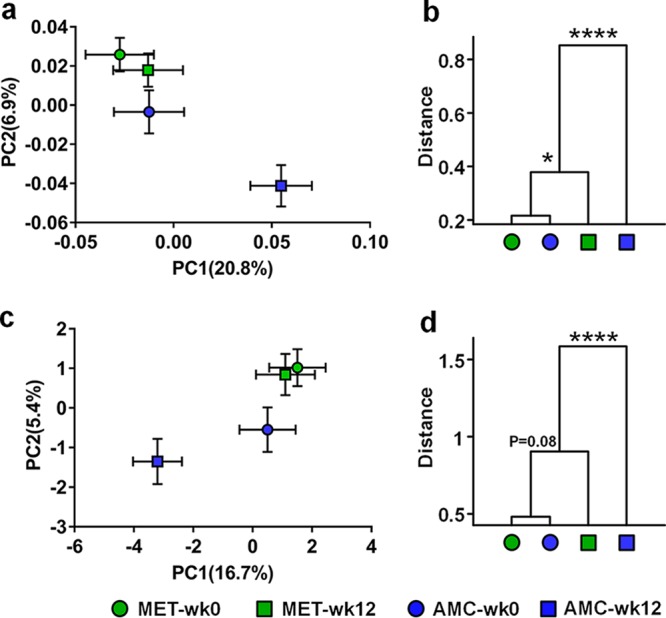
Both metformin and AMC herbal formula significantly altered the overall gut microbiota structure in T2DM patients with hyperlipidemia. (a) Principal-component analysis score plot. (c) Principal-coordinate analysis score plot based on Bray-Curtis distance. Each point represents the mean principal-coordinate score of all patients in a group at one time point, and the error bar represents the SEM. (b and d) Clustering of gut microbiota based on the distances calculated using MANOVA with PCA scores and Bray-Curtis PCoA scores. ****, *P* < 0.0001; *, *P* < 0.05.

To analyze the structural changes of gut microbiota in T2DM patients treated with metformin and the AMC herbal formula, we performed Illumina sequencing of the V3 and V4 regions of the 16S rRNA gene using fecal samples collected at weeks 0 and 12 in the metformin and AMC treatment groups. Altogether, we got 6,216,877 high-quality sequences and 507 operational taxonomic units (OTUs) from 372 samples, with an average of 16,712 ± 3,663 reads. (One sample was excluded in later analysis because only 1,891 reads were obtained.) The α diversity of the gut microbiota community showed that metformin significantly increased Simpson’s diversity index, while AMC significantly decreased rarefaction and Chao1 richness estimates, indicating that metformin significantly increased diversity, whereas AMC significantly reduced it (see Fig. S3 in the supplemental material). Principal-component analysis (PCA) and principal-coordinate analysis (PCoA) based on Bray-Curtis distance showed that both metformin and AMC significantly changed the gut microbiota structure of T2DM patients (Fig. 2). As for the PCoA results based on weighted and unweighted UniFrac distances and abundance-weighted and binary Jaccard distances, they displayed similar values, although the effect of metformin did not reach a significant level (see Fig. S4 in the supplemental material). Furthermore, comparing the changes of five distances before and after treatments using the Mann-Whitney *U* test, AMC exerted a significant larger modulating effect on the gut microbiota structure (see Fig. S5 in the supplemental material).

10.1128/mBio.02392-17.4 FIG S3  Effects of metformin and AMC herbal formula treatment on the diversity of the gut microbiota. (a) OTU estimates via rarefaction analysis. (b) Chao1 richness estimates. (c) Shannon-Wiener index. (d) Simpson’s index. Wilcoxon’s test is performed for comparison between the before and after treatment groups using the diversity calculation results under the same sequencing depth of 5,010. Data are presented as means ± SEM. *, *P* < 0.05; **, *P* < 0.01; ***, *P* < 0.001. Download FIG S3, TIF file, 0.6 MB. Copyright © 2018 Tong et al. 2018 Tong et al. This content is distributed under the terms of the Creative Commons Attribution 4.0 International license.

10.1128/mBio.02392-17.5 FIG S4  Overall structural alterations of gut microbiota by metformin and AMC herbal formula treatments in type 2 diabetic patients. (a, c, e, and g) Principal-coordinate analysis score plot based on weighted and unweighted UniFrac distances and abundance-weighted and binary Jaccard distances. Each point represents the mean principal-coordinate score of all patients in a group at one time point, and the error bar represents the SEM. (b, d, f, and h) Clustering of gut microbiota based on the distances calculated using MANOVA with weighted and unweighted UniFrac distances and abundance-weighted and binary Jaccard PCoA scores. ***, *P* < 0.0001. Download FIG S4, TIF file, 0.2 MB. Copyright © 2018 Tong et al. 2018 Tong et al. This content is distributed under the terms of the Creative Commons Attribution 4.0 International license.

10.1128/mBio.02392-17.6 FIG S5  Larger alteration of gut microbiota structure by AMC herbal formula than by metformin in type 2 diabetic patients. (a and b) Weighted and unweighted UniFrac distances. (c) Bray-Curtis distance. (d and e) Abundance-weighted and binary Jaccard distances. MET, metformin; AMC, AMC herbal formula. The box extends from the 25th to 75th percentiles. The upper (or lower) whisker extends from the top of the box to the highest (or lowest) value within 1.5 times the interquartile range. *, *P* < 0.05, and **, *P* < 0.01, using the Mann-Whitney *U* test. Download FIG S5, TIF file, 0.6 MB. Copyright © 2018 Tong et al. 2018 Tong et al. This content is distributed under the terms of the Creative Commons Attribution 4.0 International license.

### CAGs associated with the improvements in glucose and lipid homeostasis.

**FIG 3:**
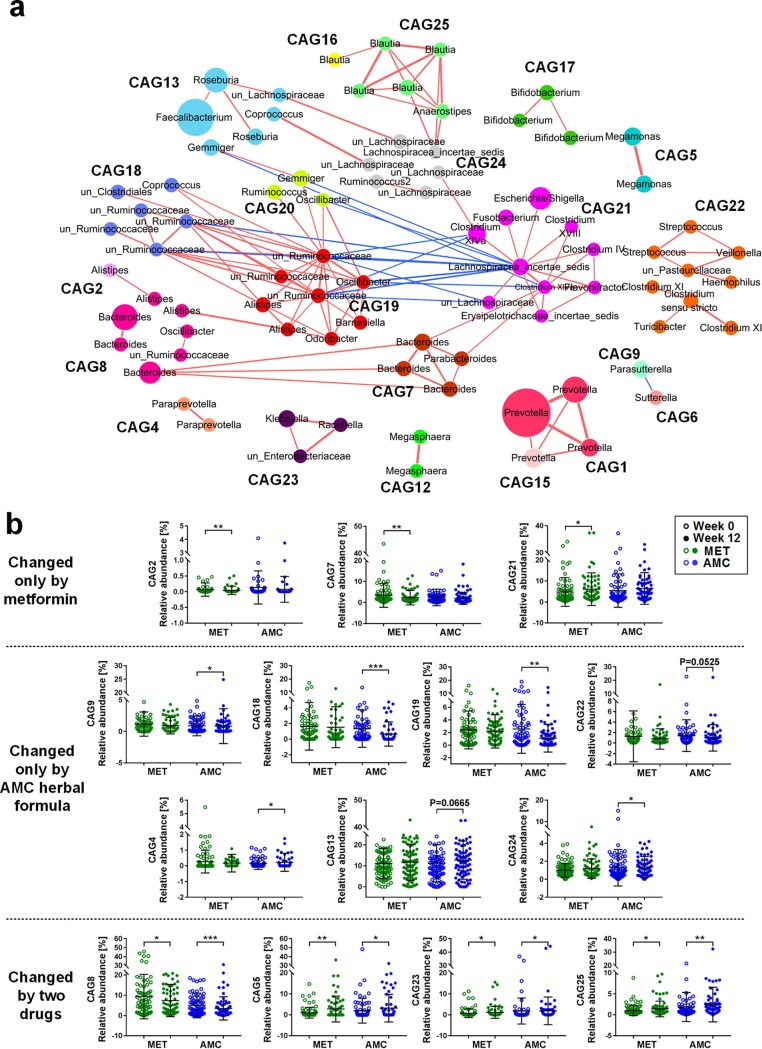
Alterations of coabundant groups by metformin and AMC herbal formula treatments. (a) Coabundant network analysis. Each circle represents one OTU. Circles with the same color are from the same coabundant group (CAG). Circle size represents the average abundance of each OTU in each group. (b) Relative abundance of altered CAGs by metformin and/or the AMC herbal formula. The relative abundances of CAGs are expressed as means ± standard deviations (SD). ***, *P* < 0.001, **, *P* < 0.01, and *, *P* < 0.05, by Wilcoxon’s signed-rank test.

To find the key phylotypes that correlated with the efficacy of metformin and the AMC herbal formula, coabundant network analysis was used in this study. The 214 core OTUs (which existed in >20% samples) were clustered into 24 coabundant groups (CAGs) by SparCC correlation and permutational multivariate analysis of variance (PERMANOVA). Twenty-one CAGs containing OTUs with *R* values of >0.4 were displayed in the coabundant network (Fig. 3a).

**FIG 4:**
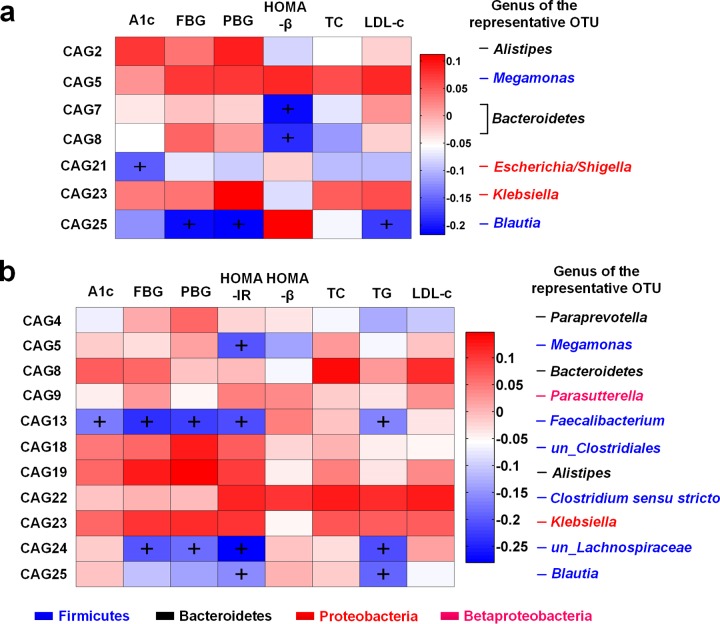
Association of the key CAGs changed by metformin or the AMC herbal formula with improvements in glucose and lipid homeostasis. (a) Key CAGs associated with the efficacy of metformin. (b) Key CAGs associated with the efficacy of the AMC herbal formula. The *R* value is shown as a heat map. +, *P* < 0.05 by Spearman’s correlation. The names of genera of the representative OTUs of each CAG are labeled to the right.

In the metformin treatment group, four CAGs were increased by metformin and three were significantly decreased (Fig. 3b; see Table S3 in the supplemental material). Among the four CAGs enriched by metformin, CAG21 showed significant inverse correlations with HbA1c, and CAG25 with FBG, 2-h PBG, and LDL-c (Fig. 4a). CAG21 consisted of 10 OTUs: two OTUs belong to *Clostridium* XIVa and one to each of the following genera: Erysipelotrichaceae incertae *sedis*, *Escherichia*/*Shigella*, *Fusobacterium*, *Flavonifractor*, *Lachnospiraceae*, Lachnospiracea incertae *sedis*, and *Clostridium* XVIII and IV. CAG25 contained four OTUs from *Blautia* and one from *Anaerostipes*. Among the three CAGs inhibited by metformin, CAG7 and CAG8 were significantly correlated with the alleviation of hyperglycemia (Fig. 4a). CAG7 and -8 showed significantly negative correlations with HOMA-β. CAG7 contained three OTUs from *Bacteroides* and one from *Parabacteroides*; CAG8 consisted of three OTUs from *Bacteroides*, two OTUs from *Alistipes*, and one OTU each from *Oscillibacter* and un-*Ruminococcaceae*, respectively. To sum up, metformin significantly enriched CAG21 and CAG25 and inhibited CAG7 and CAG8, which were significantly correlated with the amelioration of hyperglycemia and hyperlipidemia.

10.1128/mBio.02392-17.9 TABLE S3  Taxonomy and relative abundance of the 14 key CAGs. Download TABLE S3, DOCX file, 0.1 MB. Copyright © 2018 Tong et al. 2018 Tong et al. This content is distributed under the terms of the Creative Commons Attribution 4.0 International license.

After the AMC herbal formula treatment, six CAGs were increased and five were decreased (Fig. 3b; Table S3). Among the six CAGs enriched by AMC herbal formula, CAG5, CAG13, CAG24, and CAG25 showed significant positive correlations with the improvement of glucose and lipid homeostasis (Fig. 4b). CAG5 was inversely correlated with HOMA-IR, which contained two OTUs from *Megamonas*. CAG13 has the highest relative abundance, with OTU2 from *Faecalibacterium* accounting for over 50%. It showed significant negative correlations with HbA1c, FBG, 2-h PBG, HOMA-IR, and triglyceride levels, which consisted of two OTUs from *Roseburia*, and one OTU each from *Faecalibacterium*, *Gemmiger*, *Coprococcus*, and un-*Lachnospiraceae*, respectively. CAG24 also showed inverse correlations with FBG, 2-h PBG, HOMA-IR, and triglyceride levels, which contained two OTUs from un-*Lachnospiraceae* and one OTU from *Ruminococcus* 2 and Lachnospiracea incertae *sedis*, respectively. CAG25 was inversely correlated with HOMA-IR and triglyceride levels, and it contained four OTUs from *Blautia* and one from *Anaerostipes*, as described above. Taken together, AMC herbal formula significantly enriched CAG5, CAG13, CAG24, and CAG25, which showed significant correlations with the improvements of hyperglycemia and hyperlipidemia.

We compared the key CAGs that responded to metformin and the AMC herbal formula and tried to find the common CAGs shared by the two drugs. These CAGs might be related to the alleviation of diabetes rather than simply responding to the drug treatment. Among the eight CAGs that were associated with the efficacy of metformin or the AMC herbal formula, CAG25, represented by the OTU from *Blautia*, was identified in both drug treatment groups.

## DISCUSSION

To our knowledge, this is the first randomized clinical trial evaluating a traditional Chinese herbal formula’s efficacy in treating T2DM with hyperlipidemia and obesity using metformin as a positive control. In the 12 weeks of clinical trial, both drugs significantly ameliorated hyperglycemia and hyperlipidemia, but only AMC significantly improved triglyceride levels. This is consistent with a meta-analysis of randomized controlled clinical trials in which metformin had no intrinsic effect on triglyceride levels in T2DM patients (30). To sum up, AMC displayed a slightly better effect in the alleviation of hyperglycemia and hyperlipidemia as only AMC significantly improved diabetic patients’ HOMA-IR and plasma triglyceride levels.

In this clinical trial, an alteration of gut microbial structure by metformin in humans was observed. Metformin treatment significantly inhibited two functional groups, namely, CAG7 and CAG8, which contained potential pathogen-like genera, including *Alistipes*, *Oscillibacter*, and *Bacteroides*. In addition, the reductions in the two CAGs significantly correlated with the improvement of glucose homeostasis by metformin. Members of *Alistipes* were positively associated with the colonic tumorigenesis in gnotobiotic mice into which were transplanted the gut microbiota from colorectal cancer patients (31). Furthermore, elevation of several OTUs from *Alistipes* was correlated with a greater frequency of pain in pediatric patients with irritable bowel syndrome (32). As for *Oscillibacter*, transferring the gut microbiota of obesity-prone rats harboring specific species from *Oscillibacter* and *Clostridium* clusters IV to germfree mice replicated the obese phenotypes and associated disordered signaling pathways (33). These results suggest that inhibition of potential pathogen-like bacteria might be involved in the glucose-lowering effect of metformin.

The present study observed the increase in *Blautia* spp., a population of SCFA producers in the metformin-treated group, in agreement with our previous animal study (18). Interestingly, we observed a decrease in *Akkermansia* in T2DM patients after metformin treatment. This finding confirmed our results in Wistar rats with HFD-induced insulin resistance and obesity treated with metformin (18), but it was contrary to the previous study that metformin specifically increased *Akkermansia* spp. and thus improved glucose homeostasis in mice with HFD-induced obesity (17). This might be attributed to the differences in strain-specific functions. To further dissect the gut microbiota-mediated mechanism of metformin in the later study, we first tried to identify the key population that responds to metformin in a randomized clinical trial.

AMC increased functional groups CAG5 and CAG13, which were significantly correlated with the improvements in glucose and lipid homeostasis. They contained OTUs from *Faecalibacterium*, *Roseburia*, *Gemmiger*, *Coprococcus*, and *Megamonas*. *Faecalibacterium* and *Roseburia* are two genera containing butyrate-producing bacteria (34, 35). Faecalibacterium prausnitzii is an anti-inflammatory (36) and functionally important bacterium (37). Several recent studies showed that F. prausnitzii and its supernatant prevented the physiological damage to the intestine induced by 2,4,6-trinitrobenzenesulfonic acid (TNBS) (36, 38) or dextran sodium sulfate (DSS) (39) in mice, possibly through the production of butyrate, salicylic acids (40), or an anti-inflammatory molecule (41). The gut metagenome analysis showed that T2DM patients had lower levels of butyrate-producing bacteria, such as *Roseburia* and F. prausnitzii, in the cohorts of Chinese diabetics and European diabetic women (42–44). It has been reported that a higher abundance of F. prausnitzii was linked with the reduction of a low-grade inflammatory state during the alleviation of obesity and diabetes in patients receiving Roux-en-Y gastric bypass (RYGB) surgery (45). Moreover, according to our previous study, an increase in F. prausnitzii was associated with the alleviation of diabetes by another Chinese herbal formula, GQD, in a double-blinded, placebo-controlled clinical trial (20). *Faecalibacterium* was also recovered after the treatment of AMC in this study, indicating that *Faecalibacterium* spp. might be important in the efficacy of the TCM formula in the improvements in diabetes and obesity.

*Roseburia*, *Gemmiger*, and *Coprococcus* enriched by the AMC herbal formula in T2DM patients are also reported to have beneficial influences. Besides, restoration of *Roseburia* spp. by chitin-glucan fiber was related with alleviations of obesity, diabetes, and hepatic steatosis in mice fed with HFD (46). These results showed that enrichment of SCFA-producing bacteria might be associated with the amelioration of hyperglycemia and hyperlipidemia by AMC.

Notably, CAG25 dominated by OTUs from *Blautia* was significantly increased by both drugs and correlated with the improvements in glucose and lipid homeostasis. *Blautia* is a group of bacteria containing various acetate and butyrate producers (47, 48). More importantly, *Blautia* spp. were also reported to be enriched by metformin during the amelioration of obesity and insulin resistance in rats (18). It has been reported that 16 children with type 1 diabetes possessed a significantly lower abundance of Blautia coccoides with respect to 16 healthy children by PCR-denaturing gradient gel electrophoresis (DGGE) and real-time quantitative PCR (qPCR) (49). Moreover, according to the gut microbial structure analysis of 64 patients receiving blood/marrow transplantation, a larger amount of anti-inflammatory *Blautia* spp. was associated with a reduction in mortality caused by acute graft-versus-host disease after the surgery. This result was further confirmed in another independent cohort with 51 patients enrolled from the same institution (50). These results suggest that *Blautia* spp. might be a common target for the management of diabetes with hyperlipidemia.

Compared with metformin, AMC exerted a much stronger modulatory effect on the gut microbiota together with better efficacy in improving HOMA-IR and triglyceride levels. This might be attributed to the effects of multiple chemical components in the AMC, such as mangiferin and berberine. In a randomized, double-blind clinical trial, mangiferin treatment significantly improved serum levels of triglycerides, free fatty acids, and HOMA-IR in overweight patients with hyperlipidemia (51), but the pharmacological mechanism of mangiferin has not been elucidated yet. Another study showed that gut microbiota might play an important role in it despite its poor absorption in the gastrointestinal tract. *Bacteroides* sp. strain MANG might be responsible for the pharmacological effects of mangiferin via converting it into its aglycone and norathyriol (52). Berberine was reported to significantly improve hyperglycemia and hyperlipidemia in a multicenter, randomized, double-blind, and placebo-controlled clinical trial (24). In addition, berberine prevented and alleviated HFD-induced obesity and insulin resistance in rats through enrichment of SCFA producers such as *Blautia* spp. (18, 29). These findings implied that mangiferin and berberine might be the major active ingredients in AMC that shifted the gut microbiota.

### Conclusion.

Taken together, these data suggest that metformin and the Chinese herbal formula may ameliorate type 2 diabetes with hyperlipidemia by enriching beneficial bacteria, such as *Blautia* and *Faecalibacterium* spp.

## MATERIALS AND METHODS

### Study design.

This study was a multicenter, randomized, positive-control, and open label clinical trial, including a 4-week washout period and a 12-week treatment period. It was performed according to the Declaration of Helsinki (2008) and approved by the Ethics Committee of Guang’anmen Hospital of the China Academy of Chinese Medical Sciences (approval no. eighty-sixth 2011). All participants signed informed consent forms before enrolling in the study (Clinicaltrials.gov registration no. NCT01471275). The inclusion and exclusion criteria are stated in Text S1 in the supplemental material.

10.1128/mBio.02392-17.1 TEXT S1  Supplemental materials and methods. Download TEXT S1, DOCX file, 0.1 MB. Copyright © 2018 Tong et al. 2018 Tong et al. This content is distributed under the terms of the Creative Commons Attribution 4.0 International license.

In the initial screening, a 75-g oral glucose tolerance test (OGTT) was used for diagnosis of diabetes, and plasma triglyceride levels, waist circumference, and hip circumference were used for diagnosis of hyperlipidemia. After a 4-week washout period, 450 patients with T2DM and hyperlipidemia were enrolled in the clinical trial. They were randomized into two treatment groups, receiving either metformin or TCM. Randomization was performed centrally and stratified in the proportion of 1:1 for the two treatment groups through the Interactive Web Response System by a third party not involved in data management and statistical analysis. After a 12-week treatment, 100 patients were randomly chosen from either treatment group. The random assignment was generated by the computer program SAS 9.3, which was also conducted by independent statisticians who did not participate in data acquisition and evaluation. All of the recruited patients followed advice on diabetic and low-fat diets and exercises for diabetic patients as instructed by the Chinese Diabetes Society.

### Drug administration.

The TCM formula in our study was the AMC herbal formula composed of 8 herbs, namely, *Rhizoma Anemarrhenae*, Momordica charantia, Coptis chinensis, Salvia miltiorrhiza, red yeast rice, Aloe vera, Schisandra chinensis, and dried ginger. Herbs were all provided by Jiangyin Tianjiang Pharmaceutical Co., Ltd., and quality controlled. All the herbs were processed into boil-free granules (see Text S1 and Fig. S1 in the supplemental material). Table S1 in the supplemental material lists the amount of granular extracts of each herb in 1 unit of AMC formula the patients had daily. Patients mixed the eight granular extracts and took them like coffee twice daily.

Patients in the positive-control group took metformin hydrochloride tablets, which were all provided by Beijing Liling Hengtai Pharmaceutical Co., Ltd., and quality controlled (approval no. H11021560). They took metformin tablets 0.25 g/time and 3 times/day orally after meals.

### Study evaluation and outcomes.

The primary efficacy outcomes were glycosylated hemoglobin (HbA1c), triglyceride (TG), weight, and waist circumference. Secondary efficacy outcomes were fasting blood glucose (FBG), 2-h postprandial blood glucose (2-h PBG), serum insulin, total cholesterol, high-density lipoprotein cholesterol (HDL-c), and low-density lipoprotein cholesterol (LDL-c). The adverse events and the occurrence of hypoglycemia were recorded. FBG, weight, and waist circumference were detected every 4 weeks; other assessments were performed before and after the treatment. Fecal samples were collected by using InviMag stool collection tubes with DNA stabilizer (Invitek, Germany) for gut microbiota analysis before and after the treatment.

### DNA extraction and V3 and V4 regions of 16S rRNA gene sequencing.

Total genomic DNA of fecal samples was extracted by the InviMag stool DNA kit (Invitek, Germany) as previously described (20). The sequencing library of the V3 and V4 regions of the 16S rRNA gene was prepared following the protocol from Illumina, Inc., with minor modification (53). Before the sequencing, 40% Phix (PhiX control kit v3; Illumina, Inc.) was added to the pooled library to serve as an internal control. The pooled library was sequenced on the Illumina MiSeq System using paired-end 300-bp reads and the MiSeq v3 reagent kit.

### Bioinformatics analysis of sequencing data.

High-quality sequencing data extraction was performed as follows (54). (i) Both ends of the forward and reverse reads were truncated at the base, where the Phred quality score was no more than 20. (ii) If the forward and reverse reads had a minimum 50-bp-length overlap, they could be merged into a complete read. (iii) These reads were not kept unless they were longer than 399 bp and the expected errors were less than 0.5. Operational taxonomic units (OTUs) were delineated at the cutoff of 97% using the USEARCH v.7.0.1090 pipeline. The protocol can be found on the website http://drive5.com/usearch/manual/uparse_pipeline.html. The detailed procures were stated in our previous publication (53). α- and β-diversity analyses were performed using QIIME v1.8.0 (55). To normalize read depth, all samples were randomly subsampled to 5,000 reads/sample. Shannon’s index, Simpson’s index, rarefaction, and Chao1 estimates were evaluated. A normalized and log_10_-transformed OTU abundance table was used for the β-diversity analysis, including principal-component analysis (PCA) and principal-coordinate analysis (PCoA) based on Bray-Curtis, UniFrac, and Jaccard distances. The taxonomy of each OTU was assigned online by RDP Classifier (RDP Naive Bayesian rRNA Classifier version 2.10) with a bootstrap cutoff of 80%.

### Coabundant network analysis.

In ecologic systems, species with similar requirements and functions would constitute an ecologic guild to adapt to the environments. Species from the same guild were shown to be coabundant. In this study, coabundant network analysis was used to find the key phylotypes associated with the improvements in glucose and lipid homeostasis. The correlations between the core OTUs (which existed in more than 20% samples) were calculated with the SparCC algorithm (bootstrap value, 100) (56). The 214 core OTUs were clustered into 24 coabundant groups (CAGs) by using the ward algorithm and PERMANOVA (999 permutations; *P* < 0.001) based on the SparCC correlation coefficient matrix with R version 3.1.3. The 21 CAGs with an *R* coefficient higher than 0.4 were visualized into a network with cytoscape v3.1.1 (http://www.cytoscape.org/). The abundance of each CAG was the sum of relative abundances of the OTUs in this CAG.

### Statistical analysis.

With regard to the demographic and clinical parameter, the two-tailed *t* test was used for paired analysis (before and after intervention). ANCOVA was used to compare treatment groups with baseline values as covariates. Missing data were handled using the last observation carried forward method. As for the gut microbiota data, Wilcoxon’s signed-rank test was used for comparison of paired samples, and the Mann-Whitney *U* test was used for independent samples.

### Accession number(s).

The sequence data have been uploaded to the Sequence Read Archive database under accession no. SRP126564.

## References

[1] Aguiree F, Brown A, Cho NH, Dahlquist G, Dodd S, Dunning T, Hirst M, Hwang C, Magliano D, Patterson C, Scott C, Shaw J, Soltesz G, Usher-Smith J, Whiting D 2013, IDF diabetes atlas, 6th ed. International Diabetes Federation, Basel, Switzerland.

[2] Kahn SE, Cooper ME, Del Prato S 2014 Pathophysiology and treatment of type 2 diabetes: perspectives on the past, present, and future. Lancet 383:1068–1083. doi:10.1016/S0140-6736(13)62154-6. 24315620 PMC4226760

[3] Donath MY, Shoelson SE 2011 Type 2 diabetes as an inflammatory disease. Nat Rev Immunol 11:98–107. doi:10.1038/nri2925. 21233852

[4] Hotamisligil GS 2008 Inflammation and endoplasmic reticulum stress in obesity and diabetes. Int J Obes 32(Suppl 7):S52–S54. doi:10.1038/ijo.2008.238. PMC2885768 19136991

[5] Shoelson SE, Goldfine AB 2009 Getting away from glucose: fanning the flames of obesity-induced inflammation. Nat Med 15:373–374. doi:10.1038/nm0409-373. 19350009 PMC4097148

[6] Zhao L 2013 The gut microbiota and obesity: from correlation to causality. Nat Rev Microbiol 11:639–647. doi:10.1038/nrmicro3089. 23912213

[7] Turnbaugh PJ, Ley RE, Mahowald MA, Magrini V, Mardis ER, Gordon JI 2006 An obesity-associated gut microbiome with increased capacity for energy harvest. Nature 444:1027–1031. doi:10.1038/nature05414. 17183312

[8] Vijay-Kumar M, Aitken JD, Carvalho FA, Cullender TC, Mwangi S, Srinivasan S, Sitaraman SV, Knight R, Ley RE, Gewirtz AT 2010 Metabolic syndrome and altered gut microbiota in mice lacking Toll-like receptor 5. Science 328:228–231. doi:10.1126/science.1179721. 20203013 PMC4714868

[9] Fei N, Zhao L 2013 An opportunistic pathogen isolated from the gut of an obese human causes obesity in germfree mice. ISME J 7:880–884. doi:10.1038/ismej.2012.153. 23235292 PMC3603399

[10] Zhang C, Yin A, Li H, Wang R, Wu G, Shen J, Zhang M, Wang L, Hou Y, Ouyang H, Zhang Y, Zheng Y, Wang J, Lv X, Wang Y, Zhang F, Zeng B, Li W, Yan F, Zhao Y, Pang X, Zhang X, Fu H, Chen F, Zhao N, Hamaker BR, Bridgewater LC, Weinkove D, Clement K, Dore J, Holmes E, Xiao H, Zhao G, Yang S, Bork P, Nicholson JK, Wei H, Tang H, Zhang X, Zhao L 2015 Dietary modulation of gut microbiota contributes to alleviation of both genetic and simple obesity in children. EBioMedicine 2:968–984. doi:10.1016/j.ebiom.2015.07.007. 26425705 PMC4563136

[11] Pruski M, Krysiak R, Okopien B 2009 Pleiotropic action of short-term metformin and fenofibrate treatment, combined with lifestyle intervention, in type 2 diabetic patients with mixed dyslipidemia. Diabetes Care 32:1421–1424. doi:10.2337/dc08-2335. 19435959 PMC2713621

[12] Luong DQ, Oster R, Ashraf AP 2015 Metformin treatment improves weight and dyslipidemia in children with metabolic syndrome. J Pediatr Endocrinol Metab 28:649–655. doi:10.1515/jpem-2014-0201. 25210757

[13] Cittadini A, Napoli R, Monti MG, Rea D, Longobardi S, Netti PA, Walser M, Samà M, Aimaretti G, Isgaard J, Saccà L 2012 Metformin prevents the development of chronic heart failure in the SHHF rat model. Diabetes 61:944–953. doi:10.2337/db11-1132. 22344560 PMC3314362

[14] He L, Sabet A, Djedjos S, Miller R, Sun X, Hussain MA, Radovick S, Wondisford FE 2009 Metformin and insulin suppress hepatic gluconeogenesis through phosphorylation of CREB binding protein. Cell 137:635–646. doi:10.1016/j.cell.2009.03.016. 19450513 PMC2775562

[15] Zhou G, Myers R, Li Y, Chen Y, Shen X, Fenyk-Melody J, Wu M, Ventre J, Doebber T, Fujii N, Musi N, Hirshman MF, Goodyear LJ, Moller DE 2001 Role of AMP-activated protein kinase in mechanism of metformin action. J Clin Invest 108:1167–1174. doi:10.1172/JCI13505. 11602624 PMC209533

[16] Bonora E, Cigolini M, Bosello O, Zancanaro C, Capretti L, Zavaroni I, Coscelli C, Butturini U 1984 Lack of effect of intravenous metformin on plasma concentrations of glucose, insulin, C-peptide, glucagon and growth hormone in non-diabetic subjects. Curr Med Res Opin 9:47–51. doi:10.1185/03007998409109558. 6373159

[17] Shin NR, Lee JC, Lee HY, Kim MS, Whon TW, Lee MS, Bae JW 2014 An increase in the Akkermansia spp. population induced by metformin treatment improves glucose homeostasis in diet-induced obese mice. Gut 63:727–735. doi:10.1136/gutjnl-2012-303839. 23804561

[18] Zhang X, Zhao Y, Xu J, Xue Z, Zhang M, Pang X, Zhang X, Zhao L 2015 Modulation of gut microbiota by berberine and metformin during the treatment of high-fat diet-induced obesity in rats. Sci Rep 5:14405. doi:10.1038/srep14405. 26396057 PMC4585776

[19] Forslund K, Hildebrand F, Nielsen T, Falony G, Le Chatelier E, Sunagawa S, Prifti E, Vieira-Silva S, Gudmundsdottir V, Krogh Pedersen H, Arumugam M, Kristiansen K, Voigt AY, Vestergaard H, Hercog R, Costea PI, Kultima JR, Li J, Jorgensen T, Levenez F, Dore J, MetaHIT Consortium, Nielsen HB, Brunak S, Raes J, Hansen T, Wang J, Ehrlich SD, Bork P, Pedersen O 2015 Disentangling type 2 diabetes and metformin treatment signatures in the human gut microbiota. Nature 528:262–266. doi:10.1038/nature15766. 26633628 PMC4681099

[20] Xu J, Lian F, Zhao L, Zhao Y, Chen X, Zhang X, Guo Y, Zhang C, Zhou Q, Xue Z, Pang X, Zhao L, Tong X 2015 Structural modulation of gut microbiota during alleviation of type 2 diabetes with a Chinese herbal formula. ISME J 9:552–562. doi:10.1038/ismej.2014.177. 25279787 PMC4331591

[21] Han J, Yang N, Zhang F, Zhang C, Liang F, Xie W, Chen W 2015 Rhizoma Anemarrhenae extract ameliorates hyperglycemia and insulin resistance via activation of AMP-activated protein kinase in diabetic rodents. J Ethnopharmacol 172:368–376. doi:10.1016/j.jep.2015.05.016. 26162543

[22] Fuangchan A, Sonthisombat P, Seubnukarn T, Chanouan R, Chotchaisuwat P, Sirigulsatien V, Ingkaninan K, Plianbangchang P, Haines ST 2011 Hypoglycemic effect of bitter melon compared with metformin in newly diagnosed type 2 diabetes patients. J Ethnopharmacol 134:422–428. doi:10.1016/j.jep.2010.12.045. 21211558

[23] Virdi J, Sivakami S, Shahani S, Suthar AC, Banavalikar MM, Biyani MK 2003 Antihyperglycemic effects of three extracts from Momordica charantia. J Ethnopharmacol 88:107–111. doi:10.1016/S0378-8741(03)00184-3. 12902059

[24] Zhang Y, Li X, Zou D, Liu W, Yang J, Zhu N, Huo L, Wang M, Hong J, Wu P, Ren G, Ning G 2008 Treatment of type 2 diabetes and dyslipidemia with the natural plant alkaloid berberine. J Clin Endocrinol Metab 93:2559–2565. doi:10.1210/jc.2007-2404. 18397984

[25] Choi HC, Kim SJ, Son KY, Oh BJ, Cho BL 2013 Metabolic effects of aloe vera gel complex in obese prediabetes and early non-treated diabetic patients: randomized controlled trial. Nutrition 29:1110–1114. doi:10.1016/j.nut.2013.02.015. 23735317

[26] Kim K, Kim H, Kwon J, Lee S, Kong H, Im SA, Lee YH, Lee YR, Oh ST, Jo TH, Park YI, Lee CK, Kim K 2009 Hypoglycemic and hypolipidemic effects of processed Aloe vera gel in a mouse model of non-insulin-dependent diabetes mellitus. Phytomedicine 16:856–863. doi:10.1016/j.phymed.2009.02.014. 19303272

[27] Becker DJ, Gordon RY, Halbert SC, French B, Morris PB, Rader DJ 2009 Red yeast rice for dyslipidemia in statin-intolerant patients: a randomized trial. Ann Intern Med 150:830–839, W147–W149. doi:10.7326/0003-4819-150-12-200906160-00006. 19528562

[28] Verhoeven V, Van der Auwera A, Van Gaal L, Remmen R, Apers S, Stalpaert M, Wens J, Hermans N 2015 Can red yeast rice and olive extract improve lipid profile and cardiovascular risk in metabolic syndrome? A double blind, placebo controlled randomized trial. BMC Complement Altern Med 15:52. doi:10.1186/s12906-015-0576-9. 25879228 PMC4364089

[29] Zhang X, Zhao Y, Zhang M, Pang X, Xu J, Kang C, Li M, Zhang C, Zhang Z, Zhang Y, Li X, Ning G, Zhao L 2012 Structural changes of gut microbiota during berberine-mediated prevention of obesity and insulin resistance in high-fat diet-fed rats. PLoS One 7:e42529. doi:10.1371/journal.pone.0042529. 22880019 PMC3411811

[30] Wulffelé MG, Kooy A, de Zeeuw D, Stehouwer CD, Gansevoort RT 2004 The effect of metformin on blood pressure, plasma cholesterol and triglycerides in type 2 diabetes mellitus: a systematic review. J Intern Med 256:1–14. doi:10.1111/j.1365-2796.2004.01328.x. 15189360

[31] Baxter NT, Zackular JP, Chen GY, Schloss PD 2014 Structure of the gut microbiome following colonization with human feces determines colonic tumor burden. Microbiome 2:20. doi:10.1186/2049-2618-2-20. 24967088 PMC4070349

[32] Saulnier DM, Riehle K, Mistretta TA, Diaz MA, Mandal D, Raza S, Weidler EM, Qin X, Coarfa C, Milosavljevic A, Petrosino JF, Highlander S, Gibbs R, Lynch SV, Shulman RJ, Versalovic J 2011 Gastrointestinal microbiome signatures of pediatric patients with irritable bowel syndrome. Gastroenterology 141:1782–1791. doi:10.1053/j.gastro.2011.06.072. 21741921 PMC3417828

[33] Duca FA, Sakar Y, Lepage P, Devime F, Langelier B, Doré J, Covasa M 2014 Replication of obesity and associated signaling pathways through transfer of microbiota from obese-prone rats. Diabetes 63:1624–1636. doi:10.2337/db13-1526. 24430437

[34] Duncan SH, Hold GL, Barcenilla A, Stewart CS, Flint HJ 2002 Roseburia intestinalis sp. nov., a novel saccharolytic, butyrate-producing bacterium from human faeces. Int J Syst Evol Microbiol 52:1615–1620. doi:10.1099/00207713-52-5-1615. 12361264

[35] Duncan SH, Hold GL, Harmsen HJ, Stewart CS, Flint HJ 2002 Growth requirements and fermentation products of Fusobacterium prausnitzii, and a proposal to reclassify it as Faecalibacterium prausnitzii gen. nov., comb. nov. Int J Syst Evol Microbiol 52:2141–2146. doi:10.1099/00207713-52-6-2141. 12508881

[36] Sokol H, Pigneur B, Watterlot L, Lakhdari O, Bermudez-Humaran LG, Gratadoux J-J, Blugeon S, Bridonneau C, Furet J-P, Corthier G, Grangette C, Vasquez N, Pochart P, Trugnan G, Thomas G, Blottiere HM, Dore J, Marteau P, Seksik P, Langella P 2008 Faecalibacterium prausnitzii is an anti-inflammatory commensal bacterium identified by gut microbiota analysis of Crohn disease patients. Proc Natl Acad Sci U S A 105:16731–16736. doi:10.1073/pnas.0804812105. 18936492 PMC2575488

[37] Li M, Wang B, Zhang M, Rantalainen M, Wang S, Zhou H, Zhang Y, Shen J, Pang X, Zhang M, Wei H, Chen Y, Lu H, Zuo J, Su M, Qiu Y, Jia W, Xiao C, Smith LM, Yang S, Holmes E, Tang H, Zhao G, Nicholson JK, Li L, Zhao L 2008 Symbiotic gut microbes modulate human metabolic phenotypes. Proc Natl Acad Sci U S A 105:2117–2122. doi:10.1073/pnas.0712038105. 18252821 PMC2538887

[38] Martín R, Miquel S, Chain F, Natividad JM, Jury J, Lu J, Sokol H, Theodorou V, Bercik P, Verdu EF, Langella P, Bermúdez-Humarán LG 2015 Faecalibacterium prausnitzii prevents physiological damages in a chronic low-grade inflammation murine model. BMC Microbiol 15:67. doi:10.1186/s12866-015-0400-1. 25888448 PMC4391109

[39] Rossi O, Khan MT, Schwarzer M, Hudcovic T, Srutkova D, Duncan SH, Stolte EH, Kozakova H, Flint HJ, Samsom JN, Harmsen HJ, Wells JM 2015 Faecalibacterium prausnitzii strain HTF-F and its extracellular polymeric matrix attenuate clinical parameters in DSS-induced colitis. PLoS One 10:e0123013. doi:10.1371/journal.pone.0123013. 25910186 PMC4409148

[40] Miquel S, Leclerc M, Martin R, Chain F, Lenoir M, Raguideau S, Hudault S, Bridonneau C, Northen T, Bowen B, Bermúdez-Humarán LG, Sokol H, Thomas M, Langella P 2015 Identification of metabolic signatures linked to anti-inflammatory effects of Faecalibacterium prausnitzii. mBio 6:e00300-15. doi:10.1128/mBio.00300-15. 25900655 PMC4453580

[41] Quévrain E, Maubert MA, Michon C, Chain F, Marquant R, Tailhades J, Miquel S, Carlier L, Bermúdez-Humarán LG, Pigneur B, Lequin O, Kharrat P, Thomas G, Rainteau D, Aubry C, Breyner N, Afonso C, Lavielle S, Grill JP, Chassaing G, Chatel JM, Trugnan G, Xavier R, Langella P, Sokol H, Seksik P 2016 Identification of an anti-inflammatory protein from Faecalibacterium prausnitzii, a commensal bacterium deficient in Crohn’s disease. Gut 65:415–425. doi:10.1136/gutjnl-2014-307649. 26045134 PMC5136800

[42] Karlsson FH, Tremaroli V, Nookaew I, Bergström G, Behre CJ, Fagerberg B, Nielsen J, Bäckhed F 2013 Gut metagenome in European women with normal, impaired and diabetic glucose control. Nature 498:99–103. doi:10.1038/nature12198. 23719380

[43] Qin J, Li Y, Cai Z, Li S, Zhu J, Zhang F, Liang S, Zhang W, Guan Y, Shen D, Peng Y, Zhang D, Jie Z, Wu W, Qin Y, Xue W, Li J, Han L, Lu D, Wu P, Dai Y, Sun X, Li Z, Tang A, Zhong S, Li X, Chen W, Xu R, Wang M, Feng Q, Gong M, Yu J, Zhang Y, Zhang M, Hansen T, Sanchez G, Raes J, Falony G, Okuda S, Almeida M, LeChatelier E, Renault P, Pons N, Batto JM, Zhang Z, Chen H, Yang R, Zheng W, Li S, Yang H, Wang J, Ehrlich SD, Nielsen R, Pedersen O, Kristiansen K, Wang J 2012 A metagenome-wide association study of gut microbiota in type 2 diabetes. Nature 490:55–60. doi:10.1038/nature11450. 23023125

[44] Zhang X, Shen D, Fang Z, Jie Z, Qiu X, Zhang C, Chen Y, Ji L 2013 Human gut microbiota changes reveal the progression of glucose intolerance. PLoS One 8:e71108. doi:10.1371/journal.pone.0071108. 24013136 PMC3754967

[45] Furet JP, Kong LC, Tap J, Poitou C, Basdevant A, Bouillot JL, Mariat D, Corthier G, Doré J, Henegar C, Rizkalla S, Clément K 2010 Differential adaptation of human gut microbiota to bariatric surgery-induced weight loss: links with metabolic and low-grade inflammation markers. Diabetes 59:3049–3057. doi:10.2337/db10-0253. 20876719 PMC2992765

[46] Neyrinck AM, Possemiers S, Verstraete W, De Backer F, Cani PD, Delzenne NM 2012 Dietary modulation of clostridial cluster XIVa gut bacteria (Roseburia spp.) by chitin-glucan fiber improves host metabolic alterations induced by high-fat diet in mice. J Nutr Biochem 23:51–59. doi:10.1016/j.jnutbio.2010.10.008. 21411304

[47] Park SK, Kim MS, Bae JW 2013 Blautia faecis sp. nov., isolated from human faeces. Int J Syst Evol Microbiol 63:599–603. doi:10.1099/ijs.0.036541-0. 22544782

[48] Park SK, Kim MS, Roh SW, Bae JW 2012 Blautia stercoris sp. nov., isolated from human faeces. Int J Syst Evol Microbiol 62:776–779. doi:10.1099/ijs.0.031625-0. 21571940

[49] Murri M, Leiva I, Gomez-Zumaquero JM, Tinahones FJ, Cardona F, Soriguer F, Queipo-Ortuño MI 2013 Gut microbiota in children with type 1 diabetes differs from that in healthy children: a case-control study. BMC Med 11:46. doi:10.1186/1741-7015-11-46. 23433344 PMC3621820

[50] Jenq RR, Taur Y, Devlin SM, Ponce DM, Goldberg JD, Ahr KF, Littmann ER, Ling L, Gobourne AC, Miller LC, Docampo MD, Peled JU, Arpaia N, Cross JR, Peets TK, Lumish MA, Shono Y, Dudakov JA, Poeck H, Hanash AM, Barker JN, Perales MA, Giralt SA, Pamer EG, van den Brink MR 2015 Intestinal Blautia is associated with reduced death from graft-versus-host disease. Biol Blood Marrow Transplant 21:1373–1383. doi:10.1016/j.bbmt.2015.04.016. 25977230 PMC4516127

[51] Na L, Zhang Q, Jiang S, Du S, Zhang W, Li Y, Sun C, Niu Y 2015 Mangiferin supplementation improves serum lipid profiles in overweight patients with hyperlipidemia: a double-blind randomized controlled trial. Sci Rep 5:10344. doi:10.1038/srep10344. 25989216 PMC4437311

[52] Sanugul K, Akao T, Li Y, Kakiuchi N, Nakamura N, Hattori M 2005 Isolation of a human intestinal bacterium that transforms mangiferin to norathyriol and inducibility of the enzyme that cleaves a C-glucosyl bond. Biol Pharm Bull 28:1672–1678. doi:10.1248/bpb.28.1672. 16141538

[53] Zhang Q, Wu Y, Wang J, Wu G, Long W, Xue Z, Wang L, Zhang X, Pang X, Zhao Y, Zhao L, Zhang C 2016 Accelerated dysbiosis of gut microbiota during aggravation of DSS-induced colitis by a butyrate-producing bacterium. Sci Rep 6:27572. doi:10.1038/srep27572. 27264309 PMC4893749

[54] Edgar RC 2010 Search and clustering orders of magnitude faster than BLAST. Bioinformatics 26:2460–2461. doi:10.1093/bioinformatics/btq461. 20709691

[55] Caporaso JG, Kuczynski J, Stombaugh J, Bittinger K, Bushman FD, Costello EK, Fierer N, Peña AG, Goodrich JK, Gordon JI, Huttley GA, Kelley ST, Knights D, Koenig JE, Ley RE, Lozupone CA, McDonald D, Muegge BD, Pirrung M, Reeder J, Sevinsky JR, Turnbaugh PJ, Walters WA, Widmann J, Yatsunenko T, Zaneveld J, Knight R 2010 QIIME allows analysis of high-throughput community sequencing data. Nat Methods 7:335–336. doi:10.1038/nmeth.f.303. 20383131 PMC3156573

[56] Friedman J, Alm EJ 2012 Inferring correlation networks from genomic survey data. PLoS Comput Biol 8:e1002687. doi:10.1371/journal.pcbi.1002687. 23028285 PMC3447976

