# Supplementary figures and images for: Structural Alteration of Gut Microbiota during the Amelioration of Human Type 2 Diabetes with Hyperlipidemia by Metformin and a Traditional Chinese Herbal Formula: a Multicenter, Randomized, Open Label Clinical Trial
Source: mBio. 2018 May 22;9(3):e02392-17. doi: 10.1128/mBio.02392-17 (PMC5964358; doi:10.1128/mBio.02392-17)

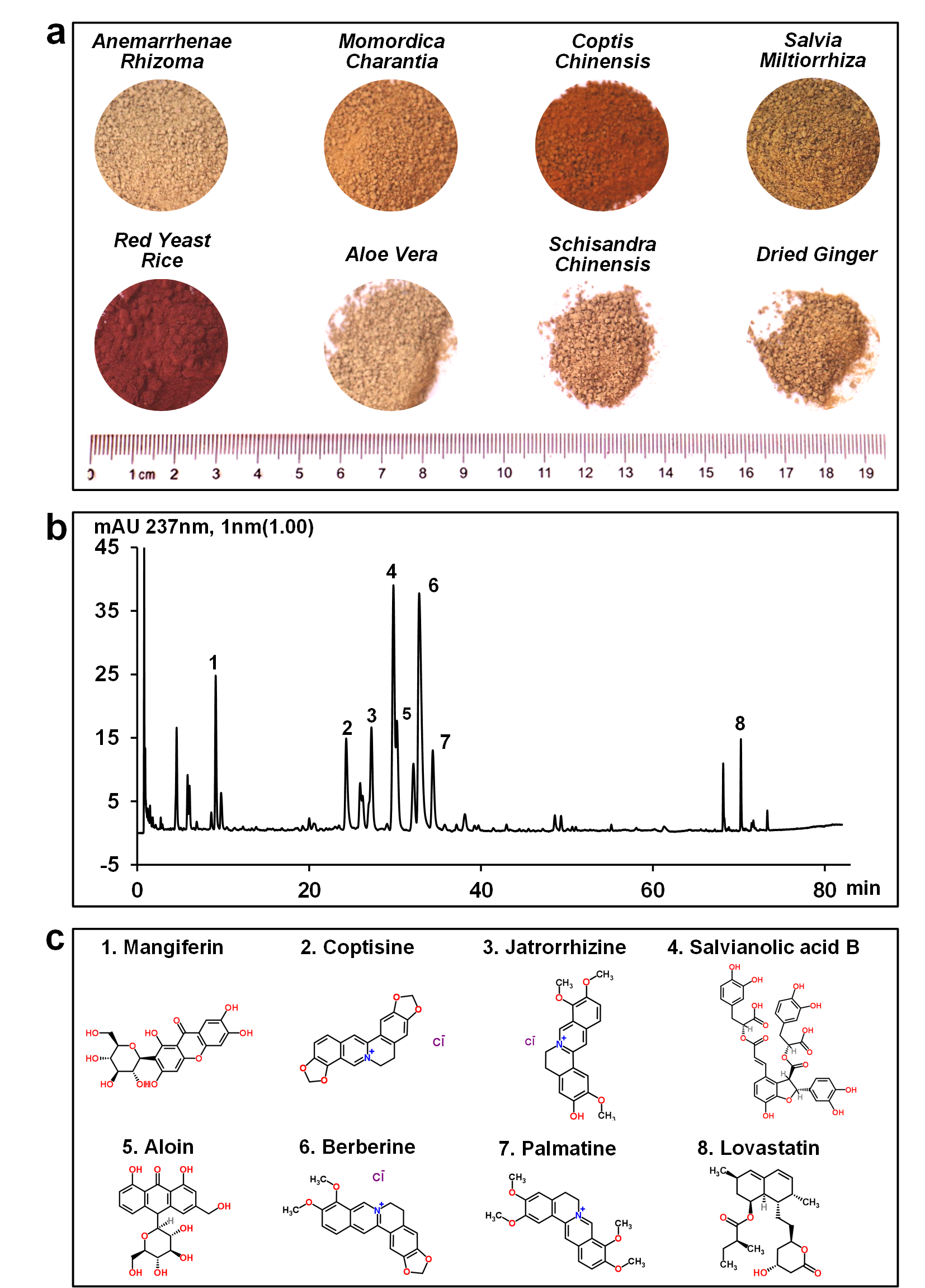

Supplement: FIG S1 [file mbo003183901sf1.tif]

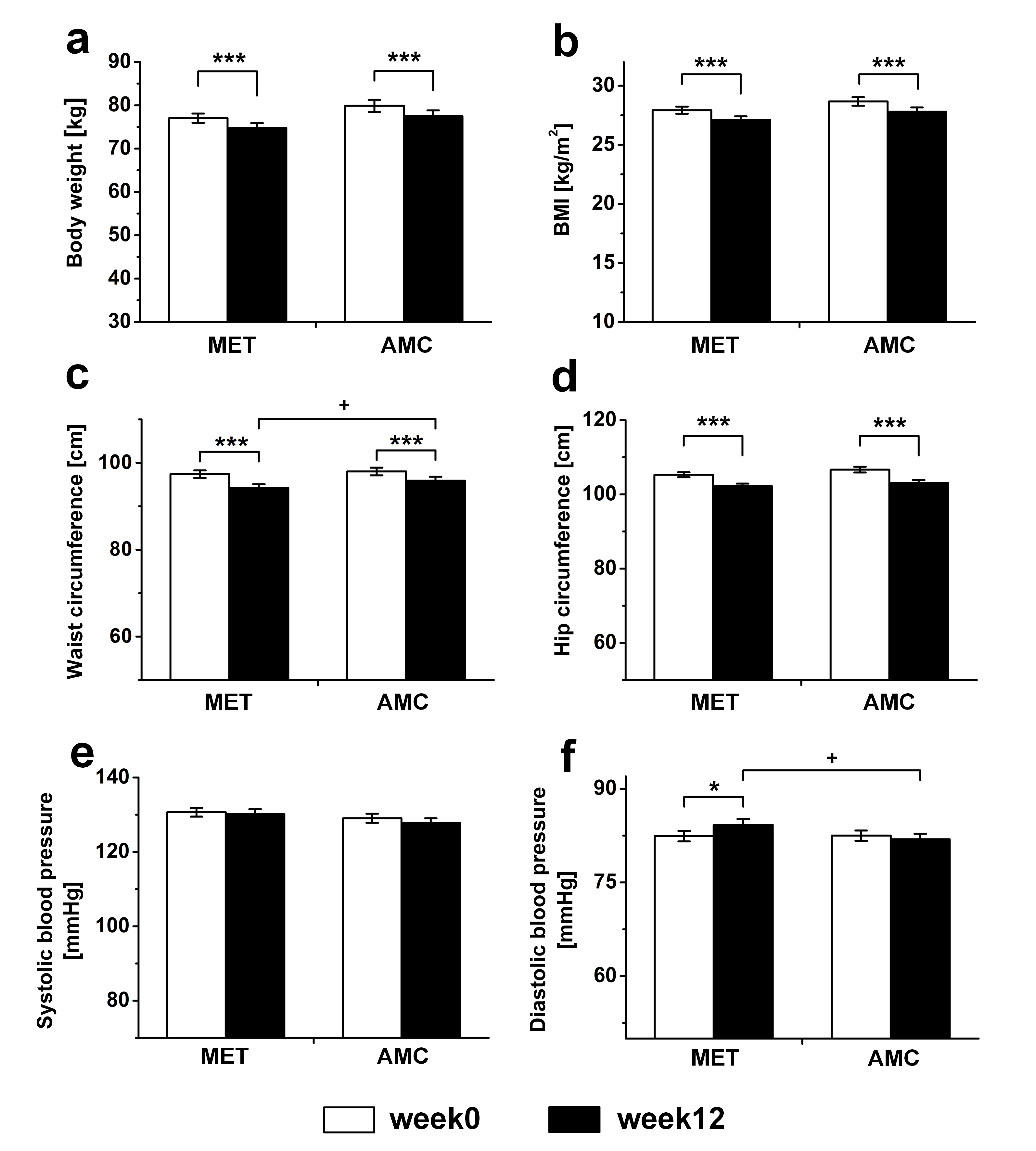

Supplement: FIG S2 [file mbo003183901sf2.tif]

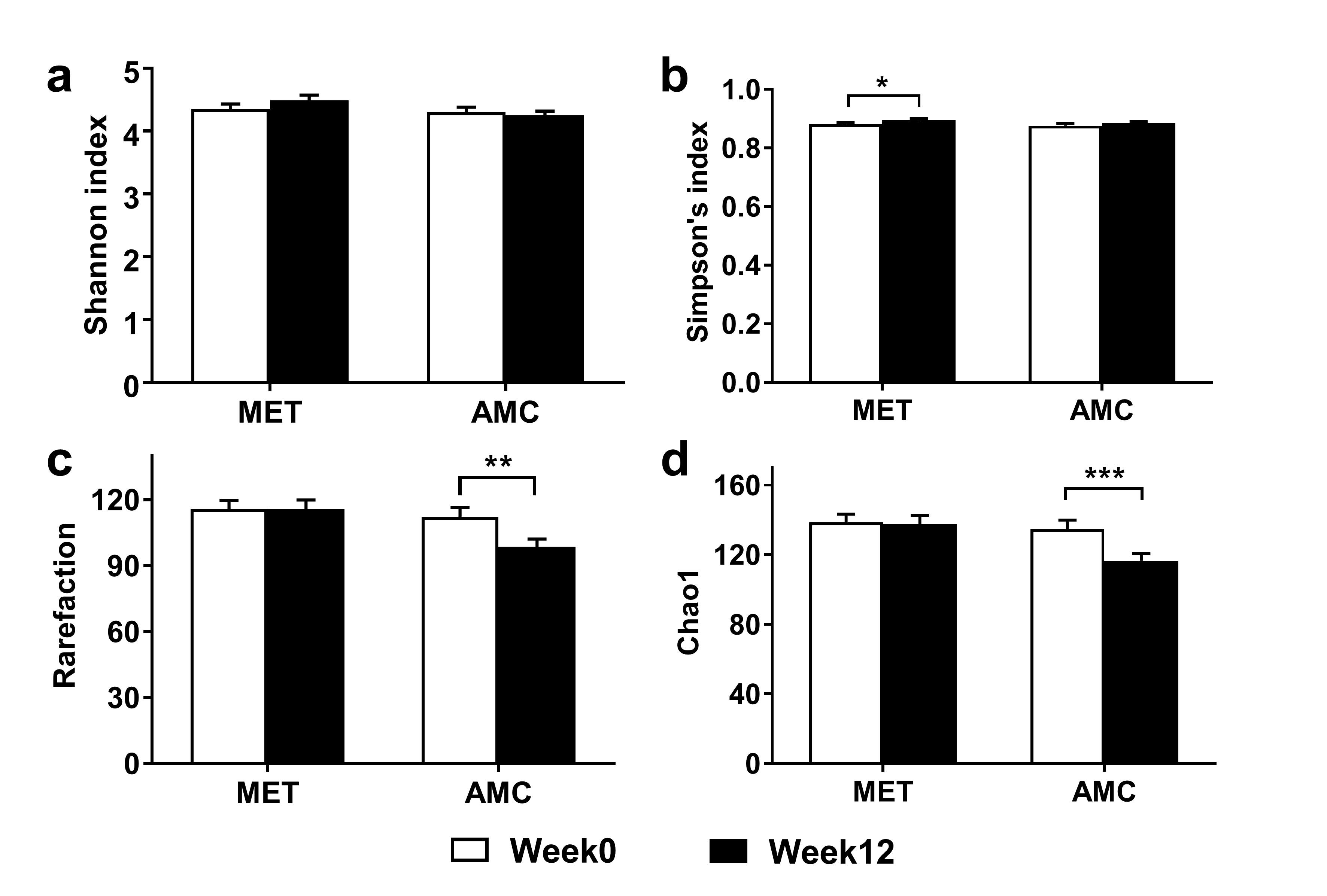

Supplement: FIG S3 [file mbo003183901sf3.tif]

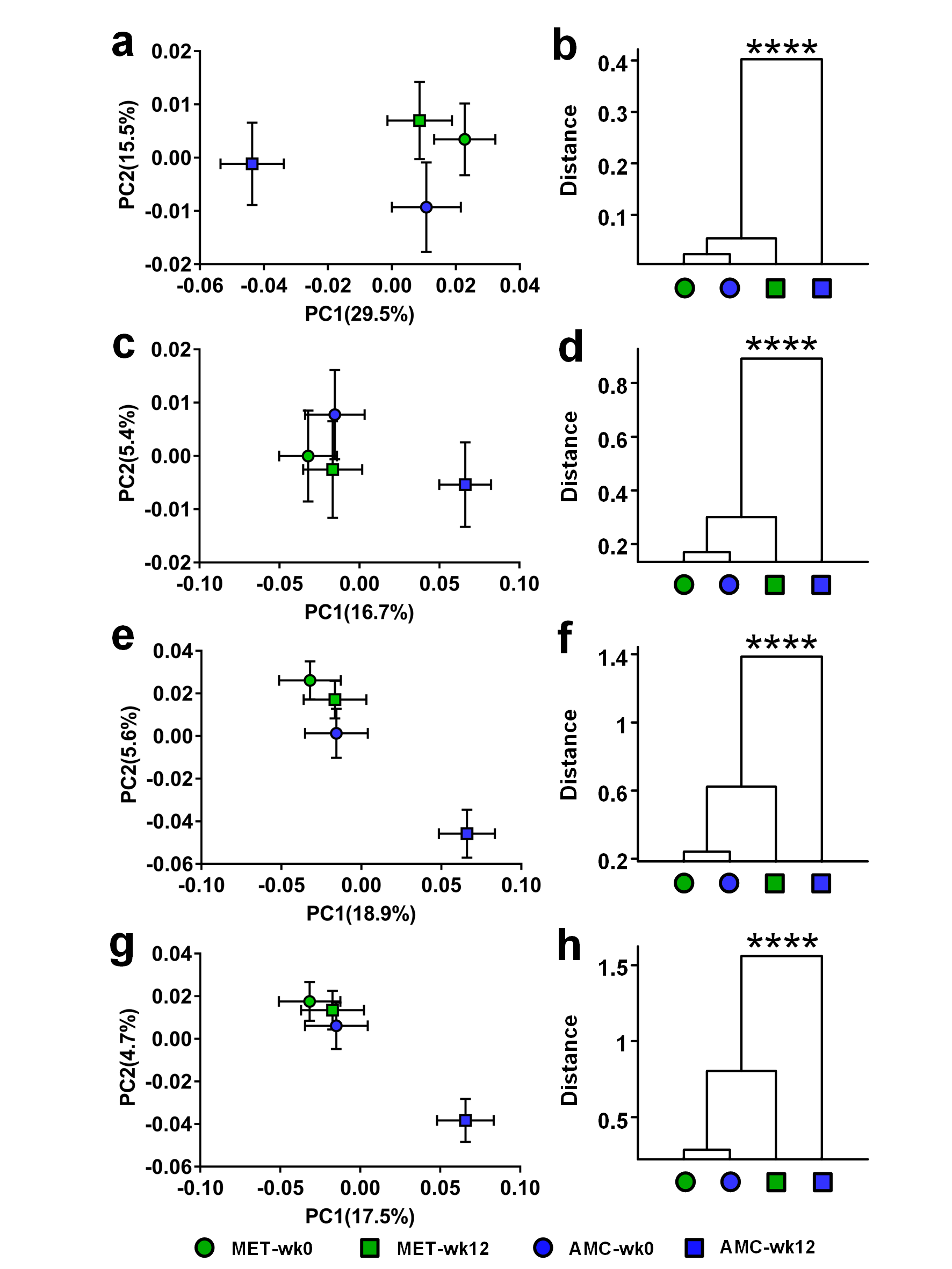

Supplement: FIG S4 [file mbo003183901sf4.tif]

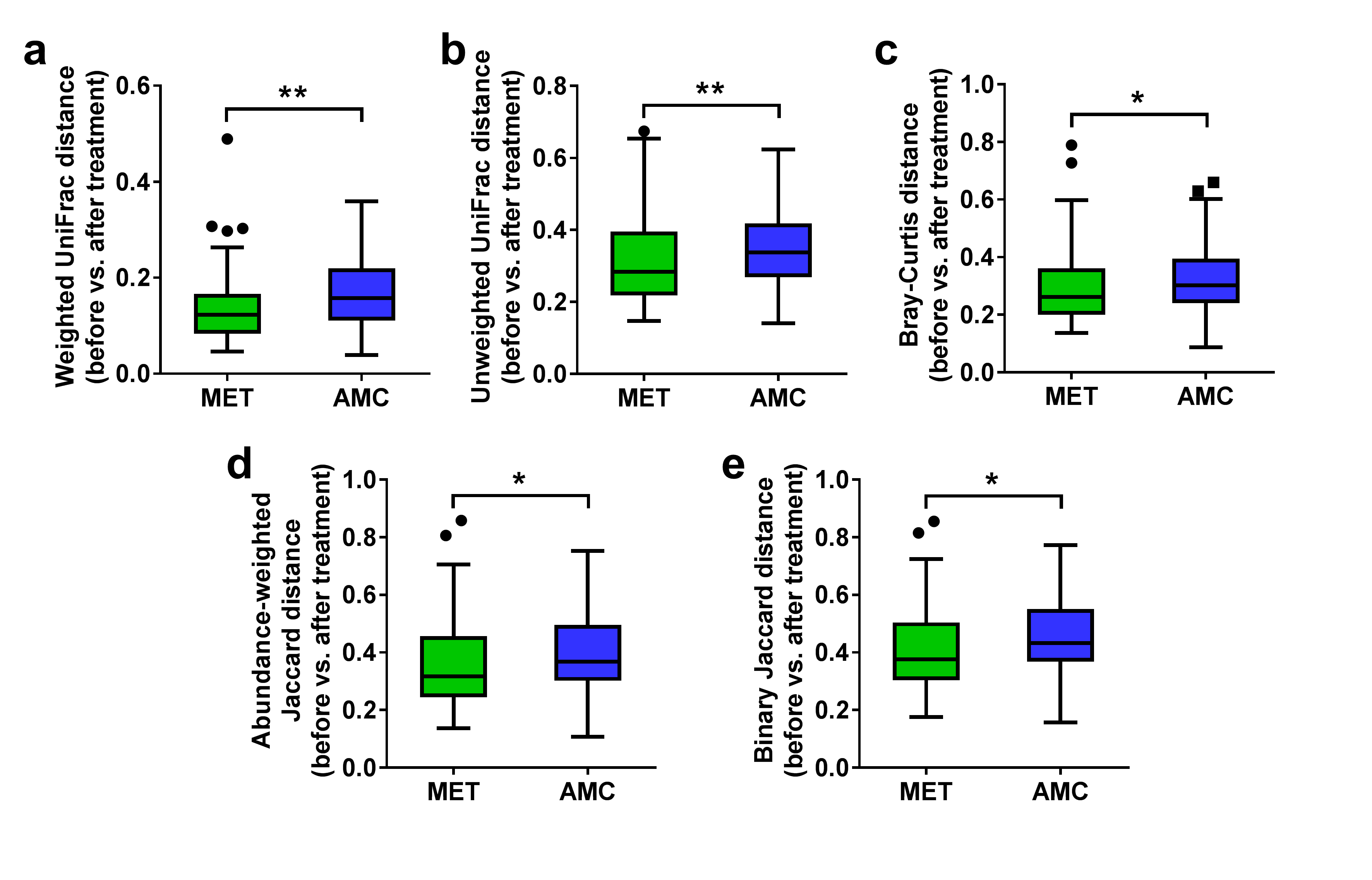

Supplement: FIG S5 [file mbo003183901sf5.tif]
