# Supplementary material for: Structural Alteration of Gut Microbiota during the Amelioration of Human Type 2 Diabetes with Hyperlipidemia by Metformin and a Traditional Chinese Herbal Formula: a Multicenter, Randomized, Open Label Clinical Trial
Source: mBio. 2018 May 22;9(3):e02392-17. doi: 10.1128/mBio.02392-17 (PMC5964358; doi:10.1128/mBio.02392-17)
Supplement: TABLE S1 [file mbo003183901st1.docx]

Table S1. The composition of AMC herbal formula.

| Herbs | Boil-free granules weight (g/day) *^a^* |
| --- | --- |
| *Anemarrhenae Rhizoma* | 7.5 |
| *Momordica Charantia* | 6 |
| *Coptis Chinensis* | 2.5 |
| *Salvia Miltiorrhiza* | 1.8 |
| *Red Yeast Rice* | 6 |
| *Aloe Vera* | 3 |
| *Schisandra Chinensis* | 1 |
| *Dried Ginger* | 1 |

*^a^* Each herb was processed into boil-free granules and packed into individual bags.
