# Supplementary material for: Structural Alteration of Gut Microbiota during the Amelioration of Human Type 2 Diabetes with Hyperlipidemia by Metformin and a Traditional Chinese Herbal Formula: a Multicenter, Randomized, Open Label Clinical Trial
Source: mBio. 2018 May 22;9(3):e02392-17. doi: 10.1128/mBio.02392-17 (PMC5964358; doi:10.1128/mBio.02392-17)
Supplement: TABLE S2 [file mbo003183901st2.docx]

Table S2. Demographic, baseline and study end point characteristics of clinical parameters.

|  | **Metformin** | | | | **AMC herbal formula** | | | | **P-value*^b^*** | **P-value*^c^*** |
| --- | --- | --- | --- | --- | --- | --- | --- | --- | --- | --- |
|  | **Week 0** | **Week 12** | | **P-value*^a^*** | **Week 0** | **Week 12** | | **P-value*^a^*** |  |  |
| No. (male/female) | 100 (50/50) | |  | | 100 (50/50) | |  | |  |  |
| Age (yr) | 58.55±9.17 | |  | | 59.00±9.46 | |  | |  |  |
| Body weight (kg) | 77.0±10.8 | 74.8±11.0 | | 0.000 | 79.9±13.9 | 77.5±13.7 | | 0.000 | 0.109 | 0.745 |
| BMI (kg/m^2^) | 27.9±3.0 | 27.1±3.0 | | 0.000 | 28.7±3.6 | 27.8±3.6 | | 0.000 | 0.118 | 0.880 |
| Waist circumference (cm) | 97.4±8.6 | 94.2±8.5 | | 0.000 | 98.0±9.0 | 95.9±9.0 | | 0.000 | 0.628 | 0.046 |
| Hip circumference (cm) | 105.3±6.7 | 102.2±6.8 | | 0.000 | 106.7±7.7 | 103.0±8.0 | | 0.000 | 0.179 | 0.398 |
| Systolic blood pressure (mm Hg) | 131±12 | 130±14 | | 0.748 | 129±12 | 128±12 | | 0.315 | 0.338 | 0.317 |
| Diastolic blood pressure (mm Hg) | 82±8 | 84±9 | | 0.037 | 82±8 | 82±9 | | 0.507 | 0.946 | 0.035 |
| HbA1c (%) | 8.13±1.12 | 7.44±1.28 | | 0.000 | 8.10±1.23 | 7.49±1.43 | | 0.000 | 0.877 | 0.700 |
| Fasting blood glucose (mmol/L) | 9.24±2.16 | 7.94±2.10 | | 0.000 | 9.81±2.69 | 8.26±2.44 | | 0.000 | 0.098 | 0.754 |
| 2h-Postprandial blood glucose (mmol/L) | 16.48±4.38 | 14.58±4.11 | | 0.000 | 16.78±4.92 | 14.47±4.76 | | 0.000 | 0.652 | 0.605 |
| Triglycerides (mmol/L) | 3.11±1.87 | 2.82±1.92 | | 0.142 | 3.34±2.17 | 2.84±1.74 | | 0.005 | 0.416 | 0.661 |
| Total cholesterol (mmol/L) | 5.59±1.04 | 5.23±1.01 | | 0.000 | 5.68±1.27 | 5.24±1.02 | | 0.000 | 0574 | 0.646 |
| HDL-c (mmol/L) | 1.19±0.27 | 1.19±0.26 | | 0.941 | 1.24±0.36 | 1.18±0.23 | | 0.068 | 0.317 | 0.290 |
| LDL-c (mmol/L) | 3.37±0.91 | 2.97±0.75 | | 0.000 | 3.31±1.08 | 3.02±0.77 | | 0.000 | 0.654 | 0.334 |
| Fasting serum insulin (μIU/mL) | 93.18±92.37 | 89.92±58.23 | | 0.721 | 107.10±66.91 | 94.80±55.38 | | 0.144 | 0.231 | 0.762 |
| HOMA-IR | 5.59±5.82 | 4.70±3.92 | | 0.160 | 6.78±5.12 | 5.15±4.16 | | 0.015 | 0.131 | 0.574 |
| HOMA-β | 51.27±54.96 | 68.55±58.83 | | 0.003 | 56.84±39.96 | 68.50±46.51 | | 0.022 | 0.421 | 0.675 |

*^a^* P value refers to comparison between week 0 vs. week 12 within each group using paired two-tailed *t*-test.

*^b^* P value refers to comparison between metformin and AMC herbal formula groups at baseline using independent two-tailed *t*-test.

*^c^* P value refers to comparison between metformin and AMC herbal formula groups after treatment using the ANCOVA analysis.

Data are presented as means ± SD.
