# Supplementary material for: Structural Alteration of Gut Microbiota during the Amelioration of Human Type 2 Diabetes with Hyperlipidemia by Metformin and a Traditional Chinese Herbal Formula: a Multicenter, Randomized, Open Label Clinical Trial
Source: mBio. 2018 May 22;9(3):e02392-17. doi: 10.1128/mBio.02392-17 (PMC5964358; doi:10.1128/mBio.02392-17)
Supplement: TABLE S3 [file mbo003183901st3.docx]

Table S3. Taxonomy and relative abundance of the 14 key CAGs

|  | Representative genus | MET_week0 | MET_week12 | AMC_week0 | AMC_week12 | P-value^a^ | |
| --- | --- | --- | --- | --- | --- | --- | --- |
|  |  |  |  |  |  | MET | AMC |
| CAG2 | *Alistipes* | 0.07±0.21 | 0.04±0.13 | 0.14±0.53 | 0.08±0.41 | 0.006 | 0.173 |
| CAG4 | *Paraprevotella* | 0.27 ± 0.72 | 0.17 ± 0.56 | 0.11 ± 0.26 | 0.23 ± 0.58 | 0.110 | 0.023 |
| CAG5 | *Megamonas* | 1.00 ± 2.56 | 2.79 ± 6.16 | 2.02 ± 5.96 | 3.26 ± 6.62 | 0.001 | 0.015 |
| CAG7 | *Bacteroidetes* | 3.45 ± 5.72 | 2.32 ± 3.46 | 2.45 ± 3.99 | 1.93 ± 2.85 | 0.011 | 0.477 |
| CAG8 | *Bacteroidetes* | 9.36 ± 10.96 | 7.39 ± 7.94 | 4.96 ± 6.12 | 3.56 ± 5.74 | 0.027 | 0.001 |
| CAG9 | *Parasutterella* | 1.17 ± 1.93 | 0.91 ± 1.30 | 0.90 ± 1.60 | 0.86 ± 2.79 | 0.341 | 0.017 |
| CAG13 | *Faecalibacterium* | 11.17 ± 7.23 | 11.73 ± 8.58 | 11.21 ± 8.94 | 12.87 ± 9.05 | 0.593 | 0.067 |
| CAG18 | *un_Clostridiales* | 1.64 ± 3.03 | 1.53 ± 2.60 | 1.35 ± 2.38 | 0.67 ± 1.55 | 0.255 | 0.000 |
| CAG19 | *Alistipes* | 2.41 ± 3.01 | 2.19 ± 2.64 | 2.55 ± 3.88 | 1.11 ± 2.20 | 0.444 | 0.006 |
| CAG21 | *Escherichia/Shigella* | 4.78 ± 6.88 | 6.11 ± 7.81 | 5.47 ± 7.99 | 6.53 ± 7.55 | 0.005 | 0.150 |
| CAG22 | *Clostridium sensu stricto* | 1.29 ± 4.85 | 0.80 ± 1.94 | 1.45 ± 3.03 | 1.01 ± 2.53 | 0.084 | 0.053 |
| CAG23 | *Klebsiella* | 0.77 ± 2.05 | 1.15 ± 2.81 | 1.82 ± 6.24 | 1.93 ± 6.64 | 0.026 | 0.028 |
| CAG24 | *un_Lachnospiraceae* | 1.01 ± 0.77 | 1.20 ± 1.08 | 1.30 ± 2.04 | 1.40 ± 1.15 | 0.248 | 0.016 |
| CAG25 | *Blautia* | 1.01 ± 1.20 | 1.41 ± 1.86 | 1.89 ± 3.47 | 2.49 ± 4.15 | 0.028 | 0.004 |

*^a^* P value refers to comparison between week 0 vs. week 12 within each group using Wilcoxon test.

Data are presented as means ± SD.
