# Supplementary material for: Structural Alteration of Gut Microbiota during the Amelioration of Human Type 2 Diabetes with Hyperlipidemia by Metformin and a Traditional Chinese Herbal Formula: a Multicenter, Randomized, Open Label Clinical Trial
Source: mBio. 2018 May 22;9(3):e02392-17. doi: 10.1128/mBio.02392-17 (PMC5964358; doi:10.1128/mBio.02392-17)
Supplement: TEXT S1 [file mbo003183901s1.docx]

## Supplementary materials and methods

**Patients Enrollment**

Participants were recruited by multi-center clinical hospitals, such as Guang’anmen hospital, from December 2011 to August 2012, and all participants signed informed consents before the study.

Inclusion criteria were: (1) informed consent was signed; (2) waistline of male ≥ 90cm, waistline of female ≥ 80cm; (3) after a screening period (diet and exercise therapy 4 weeks) newly diagnosed T2D patients according to the 1999 World Health Organization criteria (FBG 7 mmol/liter or greater and/or 2-h OGTT 11.1 mmol/liter or greater) who had not previously received pharmacological treatment; (4) HbA1c ≥ 7.0 %, FBG < 13.9 mmol/L; (5) after a screening period (diet and exercise therapy 4 weeks) TG of patients ≥1.7 mmol/L but < 5.65 mmol/L; (6) excessive heat of the stomach and the intestine for TCM syndrome; (7) age of 18-70 years old.

Exclusion criteria were: (1) patients who has been used insulin therapy or previously had a continuous period of 3 months or longer treatment of diabetes including other Chinese and Western medicine, physical therapy, psychological therapy, health food, and so on; (2) patients received hypoglycaemic or hypolipidemic medication within previous one month; (3) patients mainly with diabetic complications; or a serious heart, lung, liver, kidney and brain complications or accompanied by other serious primary diseases; (4) the systolic blood pressure ≥ 160 mmHg or diastolic blood pressure ≥ 100mmHg; (5) diabetic ketosis, diabetic ketoacidosis and severe infections within previous one month; (6) psychiatric disease; (7) pregnancy or planned pregnancy, breast-feeding women; (8) patients who were allergic to the herbal formula or its major components or allergic constitution; (9) patients attending other clinical trials present or within previous one month; (10) alcohol abuse and/or psychoactive substances, drug abuse and dependency within the past 5 years; (11) patients with the possibility of lower and complicated enrollment conditions according to the researcher’s judgment, such as Losing follow-up because of frequent changes of work environment or living environment; (12) fluctuating dosage and category of antihypertensive drugs; (13) patients taking diet pills or health food. (14) Patients with impaired liver and renal function (ALT and AST greater than 2 times the upper limit of normal; serum creatinine greater than the upper limit of normal); (15) no symptoms of hypoglycemia.

**Guidance on the diets and exercises**

All participants from the two treatment groups followed the same diet and exercise advices instructed by the Chinese Diabetes Society from the screening period to the end of the study. The daily intake calories of each patient were calculated according to his/her height, weight and physical activity. Then the doctors offered balanced dietary guidelines to the patients. Besides, all the participants were requested to take moderate-intensity exercises at least 240-480 kcal/day and 3-5 days/week.

**Standard production process of the granules of AMC herbal formula**

There are four steps for the production of boil-free granules for each herb: 1. Each herb was boiled with water for 2 times to extract the major components. 2. The decoctions of each herb were combined and filtrated with filters. 3. The filtrates of the different herbs were concentrated to pasty extracts with different relative densities under 50 ^o^C. The relative densities for *Aloe Vera*, *Coptis Chinensis*, *Rhizoma Anemarrhenae*, *Momordica Charantia*, *Salvia Miltiorrhiza*, *Schisandra Chinensis*, and *Dried Ginger* are 1.02-1.04, 1.07-1.09, 1.03-1.05, 1.03-1.05, 1.12-1.14, 1.07-1.09, and 1.06-1.08, respectively. 4. The extracts were processed to granules using spray drying and then packaged. Unlike other herbs, *Red Yeast Rice* was directly packaged since it is the powder product of yeast (*Monascus purpureus*) grown on white rice.

**Quality control of the AMC herbal formula**

All herbs used for the process of boil-free granules in this study were from the same place and in the same batch. The major chemical components of the herbs were detected and identified by Institute of Chinese Meteria Medica from China Academy of Chinese Medical Sciences. All of the herbs met the China Pharmacopoeia standard (Committee, 2010) according to the determination method in the “China Pharmacopoeia (2010 edition)”.

High performance liquid chromatography (HPLC) was performed to evaluate the stability and quality of the three batches of herbal formula granules. In brief, a reverse-phase chromolith column (4.6 mm × 100 mm; RP-18e, Merck, Germany) was used for the chromatographic separation. The mobile phase was acetonitrile-0.02% phosphoric acid contained with 0.02 mol/L potassium dihydrogen phosphate. The flow rate was 2.0 mL/min. Eight major components in the AMC herbal formula (Supplementary figure 2b and c; the structural formula of each chemical was cited from http://www.chemspider.com/) were identified at the wavelength of 237nm.
